# 2-Chloro­quinoxaline

**DOI:** 10.1107/S1600536809003717

**Published:** 2009-02-04

**Authors:** Seik Weng Ng

**Affiliations:** a Department of Chemistry, University of Malaya, 50603 Kuala Lumpur, Malaysia

## Abstract

In the title compound, C_8_H_5_ClN_2_, the planar mol­ecules are arranged with their Cl atoms in close contact [Cl⋯Cl = 3.808 (1) and 3.881 (1) Å], indicating weak Cl⋯Cl inter­actions, which give rise to a supra­molecular chain.

## Related literature

The title compound is a reagent in the synthesis of chloro­quinoxaline sulfamide, which is active against human cancers. For the synthesis of other phamaceutically active derivatives through conventional and other synthetic routes, see: Bhattacharjee *et al.* (2008 ▶); Cuenca *et al.* (2008 ▶); Hassan *et al.* (2006 ▶); Rangisetty *et al.* (2001 ▶); Rizzo *et al.* (2002 ▶); Sugimoto *et al.* (2003 ▶).
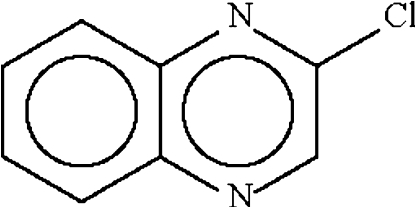


## Experimental

### 

#### Crystal data

C_8_H_5_ClN_2_
*M* *_r_* = 164.59
Monoclinic, 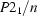

*a* = 9.1299 (2) Å
*b* = 3.8082 (1) Å
*c* = 21.0777 (6) Å
β = 93.028 (2)°
*V* = 731.82 (3) Å^3^
*Z* = 4
Mo *K*α radiation
μ = 0.44 mm^−1^
*T* = 118 (2) K
0.20 × 0.06 × 0.02 mm

#### Data collection

Bruker SMART APEX diffractometer
Absorption correction: multi-scan (*SADABS*; Sheldrick, 1996 ▶) *T*_min_ = 0.916, *T*_max_ = 0.991
6145 measured reflections
1659 independent reflections
1173 reflections with *I* > 2σ(*I*)
*R*_int_ = 0.048

#### Refinement

*R*[*F* ^2^ > 2σ(*F* ^2^)] = 0.038
*wR*(*F* ^2^) = 0.099
*S* = 1.03
1659 reflections
100 parameters
H-atom parameters constrained
Δρ_max_ = 0.29 e Å^−3^
Δρ_min_ = −0.30 e Å^−3^

### 

Data collection: *APEX2* (Bruker, 2008 ▶); cell refinement: *SAINT* (Bruker, 2008 ▶); data reduction: *SAINT*; program(s) used to solve structure: *SHELXS97* (Sheldrick, 2008 ▶); program(s) used to refine structure: *SHELXL97* (Sheldrick, 2008 ▶); molecular graphics: *X-SEED* (Barbour, 2001 ▶); software used to prepare material for publication: *publCIF* (Westrip, 2009 ▶).

## Supplementary Material

Crystal structure: contains datablocks global, I. DOI: 10.1107/S1600536809003717/tk2366sup1.cif

Structure factors: contains datablocks I. DOI: 10.1107/S1600536809003717/tk2366Isup2.hkl

Additional supplementary materials:  crystallographic information; 3D view; checkCIF report

## Figures and Tables

**Fig. 1.**
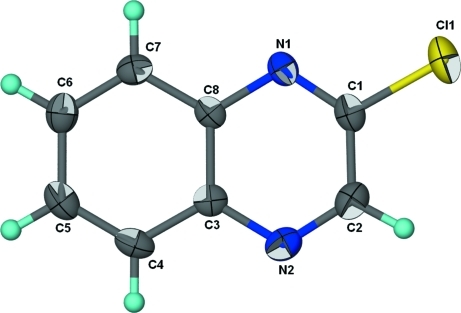
Thermal ellipsoid plot (Barbour, 2001) of C8H5ClN2; ellipsoids are drawn at the 70% probability level and H atoms of arbitrary radius.

**Fig. 2.**
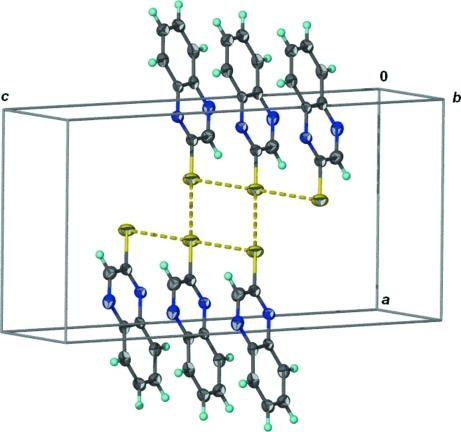
Chain structure in C8H5ClN2; the Cl···Cl contacts are shown as dashed bonds.

## supplementary crystallographic information

### Experimental

The compound was returned unchanged in an attempt at coupling it wih benzoquinone. Crystals were obtained from recrystallization from a chloroform/ether mixture.

### Refinement

Carbon-bound H-atoms were placed in calculated positions (C—H 0.93 Å) and were included in the refinement in the riding model approximation, with *U*(H) set to 1.2*U*(C).

### Crystal data

**Table:**

C_8_H_5_ClN_2_	*F*(000) = 336
*M**_r_* = 164.59	*D*_x_ = 1.494 Mg m^−^^3^
Monoclinic, *P*2_1_/*n*	Mo *K*α radiation, λ = 0.71073 Å
Hall symbol: -P 2yn	Cell parameters from 1328 reflections
*a* = 9.1299 (2) Å	θ = 2.4–28.1°
*b* = 3.8082 (1) Å	µ = 0.44 mm^−^^1^
*c* = 21.0777 (6) Å	*T* = 118 K
β = 93.028 (2)°	Prism, colorless
*V* = 731.82 (3) Å^3^	0.20 × 0.06 × 0.02 mm
*Z* = 4	

### Data collection

**Table:**

Bruker SMART APEX diffractometer	1659 independent reflections
Radiation source: fine-focus sealed tube	1173 reflections with *I* > 2σ(*I*)
graphite	*R*_int_ = 0.048
ω scans	θ_max_ = 27.5°, θ_min_ = 1.9°
Absorption correction: multi-scan (*SADABS*; Sheldrick, 1996)	*h* = −11→11
*T*_min_ = 0.916, *T*_max_ = 0.991	*k* = −4→4
6145 measured reflections	*l* = −27→27

### Refinement

**Table:**

Refinement on *F*^2^	Primary atom site location: structure-invariant direct methods
Least-squares matrix: full	Secondary atom site location: difference Fourier map
*R*[*F*^2^ > 2σ(*F*^2^)] = 0.038	Hydrogen site location: inferred from neighbouring sites
*wR*(*F*^2^) = 0.099	H-atom parameters constrained
*S* = 1.03	*w* = 1/[σ^2^(*F*_o_^2^) + (0.0465*P*)^2^ + 0.1632*P*] where *P* = (*F*_o_^2^ + 2*F*_c_^2^)/3
1659 reflections	(Δ/σ)_max_ = 0.001
100 parameters	Δρ_max_ = 0.29 e Å^−^^3^
0 restraints	Δρ_min_ = −0.30 e Å^−^^3^

### Fractional atomic coordinates and isotropic or equivalent isotropic displacement parameters (Å^2^)

**Table:**

	*x*	*y*	*z*	*U*_iso_*/*U*_eq_	
Cl1	0.60849 (6)	0.47749 (15)	0.58158 (3)	0.03308 (19)	
N1	0.88157 (18)	0.6668 (4)	0.57567 (8)	0.0202 (4)	
N2	0.8977 (2)	0.9189 (4)	0.70202 (8)	0.0248 (4)	
C1	0.7733 (2)	0.6552 (5)	0.61274 (10)	0.0219 (5)	
C2	0.7783 (2)	0.7779 (6)	0.67617 (10)	0.0261 (5)	
H2	0.6938	0.7575	0.7003	0.031*	
C3	1.0163 (2)	0.9407 (5)	0.66453 (9)	0.0194 (4)	
C4	1.1478 (2)	1.0934 (5)	0.68919 (10)	0.0231 (5)	
H4	1.1540	1.1816	0.7314	0.028*	
C5	1.2662 (2)	1.1150 (5)	0.65264 (10)	0.0250 (5)	
H5	1.3549	1.2165	0.6696	0.030*	
C6	1.2576 (2)	0.9872 (5)	0.58970 (10)	0.0254 (5)	
H6	1.3408	1.0049	0.5646	0.030*	
C7	1.1318 (2)	0.8385 (5)	0.56422 (10)	0.0214 (4)	
H7	1.1275	0.7528	0.5218	0.026*	
C8	1.0086 (2)	0.8132 (5)	0.60139 (9)	0.0179 (4)	

### Atomic displacement parameters (Å^2^)

**Table:**

	*U*^11^	*U*^22^	*U*^33^	*U*^12^	*U*^13^	*U*^23^
Cl1	0.0215 (3)	0.0305 (3)	0.0466 (4)	−0.0056 (2)	−0.0042 (2)	0.0009 (3)
N1	0.0187 (9)	0.0178 (8)	0.0239 (9)	0.0000 (7)	−0.0020 (7)	0.0008 (7)
N2	0.0286 (10)	0.0243 (9)	0.0219 (9)	0.0009 (8)	0.0049 (8)	0.0013 (7)
C1	0.0190 (10)	0.0185 (10)	0.0277 (11)	−0.0005 (8)	−0.0029 (9)	0.0043 (9)
C2	0.0252 (12)	0.0257 (11)	0.0278 (12)	0.0006 (9)	0.0067 (9)	0.0018 (10)
C3	0.0234 (10)	0.0148 (9)	0.0200 (10)	0.0022 (8)	0.0005 (8)	0.0027 (8)
C4	0.0316 (12)	0.0177 (10)	0.0194 (11)	−0.0007 (8)	−0.0047 (9)	−0.0008 (8)
C5	0.0247 (11)	0.0198 (10)	0.0296 (12)	−0.0018 (8)	−0.0066 (9)	0.0028 (9)
C6	0.0222 (11)	0.0232 (11)	0.0310 (12)	−0.0007 (9)	0.0032 (9)	0.0045 (10)
C7	0.0256 (11)	0.0192 (10)	0.0195 (10)	0.0028 (9)	0.0019 (8)	0.0000 (9)
C8	0.0188 (10)	0.0144 (9)	0.0201 (10)	0.0014 (8)	−0.0031 (8)	0.0028 (8)

### Geometric parameters (Å, °)

**Table:**

Cl1—C1	1.746 (2)	C4—C5	1.363 (3)
N1—C1	1.293 (3)	C4—H4	0.9500
N1—C8	1.372 (2)	C5—C6	1.411 (3)
N2—C2	1.308 (3)	C5—H5	0.9500
N2—C3	1.376 (3)	C6—C7	1.365 (3)
C1—C2	1.415 (3)	C6—H6	0.9500
C2—H2	0.9500	C7—C8	1.408 (3)
C3—C4	1.409 (3)	C7—H7	0.9500
C3—C8	1.415 (3)		
			
C1—N1—C8	115.61 (17)	C3—C4—H4	119.9
C2—N2—C3	116.68 (18)	C4—C5—C6	120.3 (2)
N1—C1—C2	124.93 (19)	C4—C5—H5	119.9
N1—C1—Cl1	117.21 (16)	C6—C5—H5	119.9
C2—C1—Cl1	117.87 (16)	C7—C6—C5	121.13 (19)
N2—C2—C1	120.92 (19)	C7—C6—H6	119.4
N2—C2—H2	119.5	C5—C6—H6	119.4
C1—C2—H2	119.5	C6—C7—C8	119.33 (19)
N2—C3—C4	119.62 (18)	C6—C7—H7	120.3
N2—C3—C8	121.21 (18)	C8—C7—H7	120.3
C4—C3—C8	119.18 (18)	N1—C8—C7	119.43 (18)
C5—C4—C3	120.18 (19)	N1—C8—C3	120.65 (18)
C5—C4—H4	119.9	C7—C8—C3	119.91 (18)
			
C8—N1—C1—C2	−0.5 (3)	C4—C5—C6—C7	−0.3 (3)
C8—N1—C1—Cl1	178.97 (14)	C5—C6—C7—C8	0.2 (3)
C3—N2—C2—C1	0.0 (3)	C1—N1—C8—C7	−179.61 (18)
N1—C1—C2—N2	0.6 (3)	C1—N1—C8—C3	0.0 (3)
Cl1—C1—C2—N2	−178.91 (16)	C6—C7—C8—N1	179.40 (18)
C2—N2—C3—C4	179.27 (19)	C6—C7—C8—C3	−0.2 (3)
C2—N2—C3—C8	−0.5 (3)	N2—C3—C8—N1	0.6 (3)
N2—C3—C4—C5	179.69 (18)	C4—C3—C8—N1	−179.22 (18)
C8—C3—C4—C5	−0.5 (3)	N2—C3—C8—C7	−179.87 (17)
C3—C4—C5—C6	0.5 (3)	C4—C3—C8—C7	0.3 (3)

## References

[bb1] Barbour, L. J. (2001). *J. Supramol. Chem.* **1**, 189–191.

[bb2] Bhattacharjee, G., Sondhi, S. M., Dinodia, M. & Mishra, S. K. (2008). *Ind. J. Chem. Technol.* **15**, 72–74.

[bb3] Bruker (2008). *APEX2* and *SAINT* Bruker AXS Inc., Madison, Wisconsin, USA.

[bb4] Cuenca, A., Perez, S., Yepez, A., Paredes, L., Montecinos, L., Llovera, L. & Rodriguez, C. (2008). *J. Heterocycl. Chem.* **45**, 1199–1201.

[bb5] Hassan, S. Y., Khattab, S. N., Bekhit, A. A. & Amer, A. (2006). *Bioorg. Med. Chem. Lett.* **16**, 1753–1756. 10.1016/j.bmcl.2005.11.088 16356714

[bb6] Rangisetty, J. B., Gupta, C. N. V. H. B., Prasad, A. L., Srinivas, P., Sridhar, N., Parimoo, P. & Veeranjaneyulu, A. (2001). *J. Pharm. Pharmacol.* **53**, 1409–1413. 10.1211/0022357011777765 11697550

[bb7] Rizzo, A., Campos, G., Alvarez, A. & Cuenca, A. (2002). *Synth. Commun.* **32**, 813–817.

[bb8] Sheldrick, G. M. (1996). *SADABS* University of Göttingen, Germany.

[bb9] Sheldrick, G. M. (2008). *Acta Cryst.* A**64**, 112–122. 10.1107/S0108767307043930 18156677

[bb10] Sugimoto, O., Yamada, S. & Tanji, K. (2003). *J. Org. Chem.* **68**, 2054–2057. 10.1021/jo026492a 12608838

[bb11] Westrip, S. P. (2009). *publCIF* In preparation.

